# Role of uranium toxicity and uranium-induced oxidative stress in advancing kidney injury and endothelial inflammation in rats

**DOI:** 10.1186/s40360-024-00734-w

**Published:** 2024-02-02

**Authors:** Yuwei Yang, Chunmei Dai, Xi Chen, Bin Zhang, Xiaohan Li, Wenyu Yang, Jun Wang, Jiafu Feng

**Affiliations:** 1 NHC Key Laboratory of Nuclear Technology Medical Transformation (Mianyang Central Hospital), Mianyang, 621000 P.R. China; 2 Mianyang Central Hospital, Affiliated to School of Medicine, University of Electronic Science and Technology of China, No. 12 Changjia Lane, Jingzhong Street, Mianyang, 621000 P.R. China; 3 Affiliated Hospital of Southwest Medical University, Luzhou, 646000 P.R. China; 4 College of Medical Technology, Chengdu University of Traditional Chinese Medicine, Chengdu, 611137 P.R. China

**Keywords:** Uranium toxicity, Oxidative stress, Kidney injury, Endothelial inflammation

## Abstract

**Objective:**

Uranium exposure may cause serious pathological injury to the body, which is attributed to oxidative stress and inflammation. However, the pathogenesis of uranium toxicity has not been clarified. Here, we evaluated the level of oxidative stress to determine the relationship between uranium exposure, nephrotoxic oxidative stress, and endothelial inflammation.

**Methods:**

Forty male Sprague–Dawley rats were divided into three experimental groups (U-24h, U-48h, and U-72h) and one control group. The three experimental groups were intraperitoneally injected with 2.0 mg/kg uranyl acetate, and tissue and serum samples were collected after 24, 48, and 72 h, respectively, whereas the control group was intraperitoneally injected with 1.0 ml/kg normal saline and samples were collected after 24 h. Then, we observed changes in the uranium levels and oxidative stress parameters, including the total oxidative state (TOS), total antioxidant state (TAS), and oxidative stress index (OSI) in kidney tissue and serum. We also detected the markers of kidney injury, namely urea (Ure), creatine (Cre), cystatin C (CysC), and neutrophil gelatinase-associated lipocalin (NGAL). The endothelial inflammatory markers, namely C-reactive protein (CRP), lipoprotein phospholipase A2 (Lp-PLA2), and homocysteine (Hcy), were also quantified. Finally, we analyzed the relationship among these parameters.

**Results:**

TOS (z = 3.949; *P* < 0.001), OSI (z = 5.576; *P* < 0.001), Ure (z = 3.559; *P* < 0.001), Cre (z = 3.476; *P* < 0.001), CysC (z = 4.052; *P* < 0.001), NGAL (z = 3.661; *P* < 0.001), and CRP (z = 5.286; *P* < 0.001) gradually increased after uranium exposure, whereas TAS (z = −3.823; *P* < 0.001), tissue U (z = −2.736; *P* = 0.001), Hcy (z = −2.794; *P* = 0.005), and Lp-PLA2 (z = −4.515; *P* < 0.001) gradually decreased. The serum U level showed a V-shape change (z = −1.655; *P* = 0.094). The uranium levels in the kidney tissue and serum were positively correlated with TOS (r = 0.440 and 0.424; *P* = 0.005 and 0.007) and OSI (r = 0.389 and 0.449; *P* = 0.013 and 0.004); however, serum U levels were negatively correlated with TAS (*r* = −0.349; *P* = 0.027). Partial correlation analysis revealed that NGAL was closely correlated to tissue U (*r*_*partial*_ = 0.455; *P* = 0.003), CysC was closely correlated to serum U (*r*_*partial*_ = 0.501; *P* = 0.001), and Lp-PLA2 was closely correlated to TOS (*r*_*partial*_ = 0.391; *P* = 0.014), TAS (*r*_*partial*_ = 0.569; *P* < 0.001), and OSI (*r*_*partial*_ = −0.494; *P* = 0.001). Pearson correlation analysis indicated that the Hcy levels were negatively correlated with tissue U (r = −0.344; *P* = 0.030) and positively correlated with TAS (r = 0.396; *P* = 0.011).

**Conclusion:**

The uranium-induced oxidative injury may be mainly reflected in enhanced endothelial inflammation, and the direct chemical toxicity of uranium plays an important role in the process of kidney injury, especially in renal tubular injury. In addition, CysC may be a sensitive marker reflecting the nephrotoxicity of uranium; however, Hcy is not suitable for evaluating short-term endothelial inflammation involving oxidative stress.

## Introduction

Uranium is the largest and relatively stable element and has the largest number of protons in the nuclei among the natural elements discovered to date. It is one of the important elements in the actinide family and has radioactive and chemical toxicity [1, 2]. Uranium pollution occurs through natural and depleted uranium in the environment. Industrial processes, such as ore mining, and agricultural practices, such as the application of nitrogenous or phosphate fertilizers, are the sources of natural uranium pollution, whereas military processes, such as uranium enrichment, nuclear weapon test explosions, and waste discharge from nuclear facilities, can cause depleted uranium pollution. Soil, water sources, and food sources are increasingly being impacted by both these types of uranium pollution [2, 3]. The monitoring results of the quaternary equilibrium system of Datong basin (China) and the semiarid regions of southern India revealed that uranium mainly exists in betafite or bound to carbonates and FeMn oxides in the soil, and its content ranges from 1.93 to 8.80 mg/kg. The dissolved uranium mainly exists as uranyl carbonate complexes UO_2_(CO_3_)_2_^2−^ and UO_2_(CO_3_)_3_^4−^ in the groundwater of shallow aquifers with concentrations exceeding 30 μg/L [4, 5]. Uranium contamination in the soil and groundwater ultimately leads to a higher uranium content in drinking water than that recommended by the provisional guidelines of the World Health Organization [6]. In addition, the enrichment of uranium from soil to food products, and the route of uranium exposure via food ingestion has also been proved in a few study [3]. Therefore, human health is unknowingly threatened by environmental uranium pollution.

The relationship between the radioactive effects of uranium and carcinogenesis was the first to receive attention. ^238^U, ^235^U, and ^234^U are the three isotopes of natural uranium, and ^238^U comprises >99% of uranium in nature [7]. In contrast, depleted uranium is a byproduct of the uranium enrichment process and is entirely composed of ^234^U [8]. The radioactive effect of ^238^U is extremely weak [8]; therefore, the relationship between uranium in the environment and risk of cancer occurrence is controversial [3, 9, 10]. However, uranium became a research focus in the context of environment and health in recent years because of its nephrotoxic and osteotoxic potential. Several epidemiological and clinical investigations have suggested that environmental uranium can enter the human body through the skin, digestive tract, and/or respiratory tract and exists in the human blood and other body fluids in the form of uranyl ions (UO_2_^2+^) [11–13]. Approximately 65% of the uranyl ions are filtered from the kidneys and excreted in urine within 24 h, whereas the remaining ions are deposited in tissues such as the kidneys, bones, and liver. The kidneys are the first affected organs [14, 15], and uranium accumulates at the highest levels in the kidneys. Therefore, kidneys are most sensitive to uranium intoxication, and it can induce tissue lesions and dysfunction, thereby leading to acute or chronic kidney disease.

The toxicological effects of uranium on kidneys have been confirmed in cellular and animal experiments [16, 17]. The results of animal experiments have shown that the epithelial cells of proximal tubules are primarily affected by acute uranium toxicity. The levels of blood urea nitrogen, creatinine, and urinary protein are elevated, and kidney morphological changes, such as vacuolization of kidney cells, loss of brush-like marginal membrane, and proximal tubular injury, can be observed when the cumulative content of uranium in the kidney reaches 1–3 mg/kg of tissue [18]. The results of cytotoxicity tests indicated that uranium-induced nephrotoxicity is related to electron transport chain injury and subsequent oxidative stress (OxS) in cells. Mitochondrial dysfunction may play an important role in nephrotoxicity caused by excessive exposure to uranium [19]. OxS plays an important role in the process of uranium-induced kidney cell toxicity. Conventionally, researchers have detected intracellular and peripheral blood malondialdehyde and glutathione levels or performed activity assays for one or several anti-oxidative enzymes to evaluate OxS-mediated kidney injury caused by uranium [20]. However, the components of the oxidant and antioxidant systems of the body, tissues, and cells are extremely complex. These systems act synergistically or antagonistically to regulate each other and maintain a dynamic balance [21–23]. Therefore, quantifying the changes in one or more oxides and antioxidants may not accurately reflect the OxS status of an individual. Total antioxidant status (TAS) and total oxidant status (TOS) represent the total level of antioxidants and oxidants in the body, respectively. The oxidant stress index (OSI) is the ratio of these two parameters, which reflects the balance between oxidants and antioxidants. However, TAS, TOS, and OSI have not been used to evaluate OxS in uranium-induced kidney injury. Here, we aimed to explore the correlation between the kidney toxicity of uranium and these indicators by observing the changes in uranium levels in the kidney tissue homogenate and blood, blood OxS parameters (TOS, TAS, and OSI), and biochemical markers associated with kidney injury and inflammation in rats after 24, 48, and 72 h of uranium exposure.

## Materials and methods

### Animals

Forty male healthy Sprague–Dawley rats aged 6 months (Cat No.: HR0009, Beijing HFK Bioscience Co. Ltd., CHN; body weight: 250–300 g), were maintained in a 12-h:12-h light:dark cycle at 22 °C. The rats were fed a customized chow diet (Whatman, UK) according to the American Institute of Nutrition 93 standard. The diet contained 72.7% carbohydrates, 12.5% animal proteins, and 4% fats with 18 amino acids, 13 minerals, and 13 vitamins to support the nutritional requirements of rats. Animals were divided into four groups (three experimental groups and one control group) using a numerical random table with 10 rats in each group. The experimental group rats were intraperitoneally injected with uranyl acetate dihydrate (Cat No.: U25690, Shanghai ACMEC Biochemical Technology Co. Ltd., CHN) solution at a dose of 1.0 ml/kg body weight (2.0 mg uranium per kg body weight [18]). The rats were euthanized by carbon dioxide inhalation after 24, 48, and 72 h, and the experimental groups were referred to as the U-24h, U-48h, and U-72h groups, respectively. The control (normal saline; NS) group rats were intraperitoneally injected with 1.0 ml/kg sterile physiological saline and euthanized at 24 h.

### Sample collection

Blood samples were collected from the caudal vein of adult rats in each group before euthanization and injected into SSTII separation-gel/coagulant vacutainer (Cat. No. 367955, BD, USA). After coagulation, the blood samples were centrifuged at 3000*g* for 10 min, and the serum was separated for determining uranium concentration, oxidative stress parameters, and biochemical markers related to kidney injury and inflammation. Serum samples were divided into three aliquots, and stored at −80 °C until further analysis.

We removed both kidneys from rats after euthanization. The kidneys were washed with freshly prepared phosphate-buffered saline (PBS) (pH: 7.4; Cat. No. P3813, Sigma-Aldrich, USA) and weighed. Kidney tissue was suspended in PBS (1:10 w/v) and homogenized for 1 min. The homogenate was centrifuged at 2717 g for 20 min, and the supernatant was separated for uranium quantification. Based on 1 g kidney tissue corresponding to 10 ml PBS, the tissue uranium content (Tissue U, mg/g) was 10 times the concentration of the homogenate uranium content (Homogenate U, mg/L).

### Measurement of uranium concentration

The uranium content in the serum and tissue was measured using a NexION 300Q ICP-MS (PerkinElmer, Shelton, USA). We prepared a gradient of standard uranium concentrations: 25, 50, 250, 500, and 2500 μg/L. After mixing 10 μl standard, serum or homogenate supernatant sample with 1.0 ml of 0.5% nitric acid, the strength of the nitrated sample was determined by ICP-MS. A standard curve was prepared to calculate the uranium content in the samples.

### Measurement of the oxidative stress parameters

Serum TAS and TOS levels were measured using a LAbOSPECT 008AS automatic biochemical analyzer (Hitachi, Japan). TAS determination relied on the ability of all the antioxidants in a sample to promote the reduction of ABTS+ to ABTS [2,2ʹ-azino-bis-(3-ethyl-benzothiazoline-6-sulphonate)] (Sigma-Aldrich). The TAS values were calibrated using a in-house standard of Trolox (6-hydroxy-2,5,7,8-tetramethylchroman-2-carboxylic acid; Sigma-Aldrich) and expressed as mmol/L Trolox. TOS determination relied on the ability of all the oxidants in a sample to promote the oxidation of ferrous ions to ferric ions in an acidic medium with xylenol orange (Sigma-Aldrich) as an indicator that reflected the increase in ferric ions. The TOS values were calibrated using a self-made standard of hydrogen peroxide (Chengdu United Chemical Reagents Research Institute, CHN) and expressed as μmol/L H_2_O_2_. The OxS index (OSI) was defined as the TOS-to-TAS ratio and presented in an arbitrary unit (AU). OSI (AU) = [(TOS, μmol/L H_2_O_2_)/(TAS, mmol/L Trolox] ÷ 10 [24].

### Measurement of kidney injury and endothelial inflammation biomarkers in the serum

Urea (Ure), creatinine (Cre), cystatin C (CysC), and neutrophil gelatinase-associated lipocalin (NGAL) were selected as the biomarkers of kidney injury, whereas C-reactive protein (CRP), homocysteine (Hcy), and lipoprotein phospholipase A (Lp-PLA2) were selected as the biomarkers of tissue inflammation.

Ure was detected using a urea assay kit based on the urease-glutamate dehydrogenase method (Maccura, CHN), and Cre was detected using a kit based on the sarcosine oxidase method (Maccura). Hcy levels were quantified using a kit based on the methyltransferase-coupling adenosylhomocysteinase enzymatic cycling method (Maccura), Lp-PLA2 was quantified using a 4-nitrophenol formation continuous monitoring assay kit (Maccura) and a LAbOSPECT 008AS automatic biochemical analyzer (Hitachi).

CysC levels were determined using a Mouse/Rat CysC ELISA Kit (Cat. No. E-EL-M3024, Elascience, CHN); NGAL levels were determined using a Rat NGAL ELISA Kit (Cat. No. E-EL-R0662c, Elascience), and CRP levels were determined using a Rat CRP ELISA Kit (Cat. No. E-EL-R0506c, Elascience). Each test sample and the seven standard samples provided in the kit were processed according to the manufacturer’s instructions using a Rayto 6100 enzyme marker (Rayto, China) for color detection at 450 nm. Finally, we generated second-order linear curves with the optical density values of the seven standard samples, and these curves were used to calculate the CysC, NGAL, or CRP content of each test sample.

### Statistical analysis

Statistical analysis was performed using the SPSS version 19.0 (SPSS, Chicago, USA) or MedCalc version 18.2 (MedCalc, Mariakerke, Belgium). The continuous variables in each group were certified for normal distribution using the Shapiro–Wilk test or QQ plot and were expressed as the mean plus/minus the standard deviation ($\bar x \pm s$). The one-way ANOVA test was used to analyze the differences among the U-24h, U-48h, U-72h, and NS groups, and the Bonfferoni-t test was used for pairwise comparison using the adjusted *P*-value (*Padj*; by Bonfferoni correction) <0.05 as a criterion of statistically significant difference between the two groups. The two-way ANOVA test was used to analyze the difference in the uranium levels between tissue and serum to avoid the effects of time and treatment differences. We used the Jonckheere–Terpstra test to analyze the changes in each continuous variable over time. The relationship between the tissue and serum uranium levels and the OxS parameters was analyzed and displayed using the Pearson linear correlation diagram. The absolute Pearson correlation coefficient (*r*) values of <0.200, 0.200–0.399, 0.400–0.599, 0.600–0.799, or >0.800 indicated weak, mild, moderate, strong, or extremely strong correlation, respectively [25]. The closeness of the relationship between tissue and serum uranium levels and other continuous variables was analyzed using a multivariate linear partial correlation analysis considering multicollinearity problems. The higher absolute values of the partial correlation coefficients (*r*_*partial*_) were indicative of a closer relationship among the variables. *P* or *Padj* value <0.05 was considered statistically significant.

## Results

### Uranium levels and OxS parameters in four groups of rats

Blood samples were collected from the NS group after 24 h and three experimental groups after 24, 48, and 72 h of uranium exposure, respectively. The ANOVA results indicated statistically significant differences in the levels of serum OxS parameters and serum/tissue uranium concentration among the four groups of rats (*F* = 6.254–40.621; *P* = 0.002 – <0.001) (shown in Table 1). After pairwise comparison performed by the Bonfferoni POST-HOC test, compared with the NS group, the tissue U levels (*t* = 8.868, 6.989, and 5.245; all *Padj* < 0.001) and serum U levels (*t* = 10.484, 5.468, and 7.746; all *Padj* < 0.001) increased in the U-24h, U-48h, and U-72h groups. In addition, the levels of TOS (*t* = 2.835 and 3.933; *Padj* = 0.045 and 0.002) and OSI (*t* = 3.064 and 4.255; *Padj* = 0.025 and 0.001) increased in the U-48h, U-72h, and U-72h groups, whereas the levels of TAS (*t* = −3.227 and −4.155; *Padj* = 0.016 and 0.001) decreased in these groups. We observed that changes in the OxS parameters corresponded to the changes in the tissue and serum U concentrations, thereby suggesting that uranium exposure may cause oxidative stress. In addition, compared with the U-24h group, the serum U level decreased in the U-48h group (*t* = −5.016; *Padj* < 0.001) and the OSI level increased (*t* = 2.876; *Padj* = 0.040) and the tissue U level decreased (*t* = 3.624; *Padj* = 0.005) in the U-72h group.

**Table 1:** Levels of oxidative stress parameters in the serum and uranium content in serum and tissue homogenates of rats from different groups

Biomarkers	NS (n = 10)	U-24h (n = 10)	U-48h (n = 10)	U-72h (n = 10)	*F*	*P*
TOS (μmol/L H_2_O_2_)	11.3 ± 2.91	14.41 ± 3.22	17.07 ± 4.90^a^	19.36 ± 6.78^a^	6.254	0.003
TAS (mmol/L Trolox)	1.67 ± 0.22	1.48 ± 0.17	1.33 ± 0.26^a^	1.25 ± 0.16^a^	14.196	<0.001
OSI (arbitrary unit)	0.66 ± 0.19	0.96 ± 0.21	1.35 ± 0.51^a^	1.62 ± 0.64^a,b^	23.522	<0.001
Tissue U (mg/g)	0.006 ± 0.002	23.55 ± 11.63^a^	19.49 ± 5.53^a^	14.91 ± 4.87^a,b^	30.177	<0.001
Serum U (μg/L)	0.30 ± 0.15	6.29 ± 1.54^a^	3.43 ± 1.02^a,b^	4.73 ± 1.73^a^	40.621	<0.001

*Notes* ANOVA, One-way ANOVA test. J-T test, Jonckheere-Terpstra test. Pairwise comparison by the Bonfferoni POST-HOC test, ^a^ vs. NS group, *Padj* < 0.05; ^b^ vs. U-24h group, *Padj* < 0.05. The OxS occurrence was later than the change of uranium content in tissue and serum after uranium exposure, indicating that uranium exposure may be the initiating incentive of oxidative stress

**Fig. 1:**
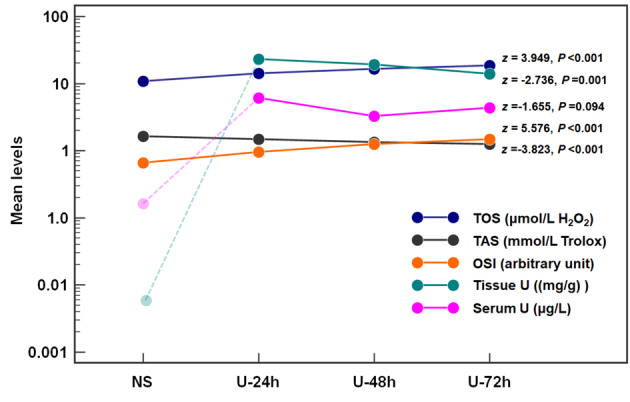
Changes in the oxidative stress parameters and serum and tissue uranium content in rats of different groups. *Notes* The circular dot represents a mean of this parameter in the corresponding group. The mean levels of Tissue U and Serum U in the NS group were excluded from the Jonckheere–Terpstra test because they did not represent the initial uranium concentration after uranium exposure. After uranium exposure, the levels of TOS and OSI show an upward trend, whereas the levels of TAS and Tissue U show a downward trend; the content of Serum U was raised at 72 h. After one-time exposure to uranium, kidney excretion and metabolism could gradually remove uranium from the body, and kidney absorption and metabolism might also participate in the excretion of uranium deposited in the kidney tissue

The Jonckheere–Terpstra test (shown in Fig. 1) indicated that the levels of TOS (*z* = 3.965; *P* < 0.001) and OSI (*z* = 5.293; *P* < 0.001) gradually increased, whereas the levels of TAS (*z* = −3.767, *P* < 0.001) gradually decreased after 72 h of uranium exposure. The tissue U levels (*z* = −2.736; *P* = 0.001) gradually decreased within 24 to 72 h. However, the Serum U levels did not exhibit a temporal trend (*z* = *−*1.655; *P* = 0.095). These results indicated that kidney excretion and metabolism could gradually clear the uranium deposited in the tissues over time after one-time exposure; however, the Serum U levels were raised at 72 h. We speculated that kidney absorption and metabolism also participated in the excretion of deposited uranium in tissues. However, the OxS that was already induced was not alleviated with the decrease in the uranium levels; notably, the OxS levels gradually increased over time.

### Interrelationship of the tissue/serum uranium content and the OxS levels

**Fig. 2:**
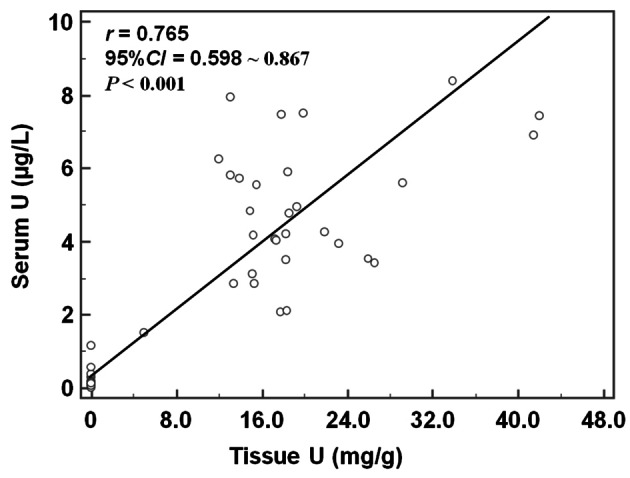
Correlation between tissue homogenate U and serum U contents. *Notes* A positive correlation existed between tissue homogenate U and serum U contents

**Fig. 3:**
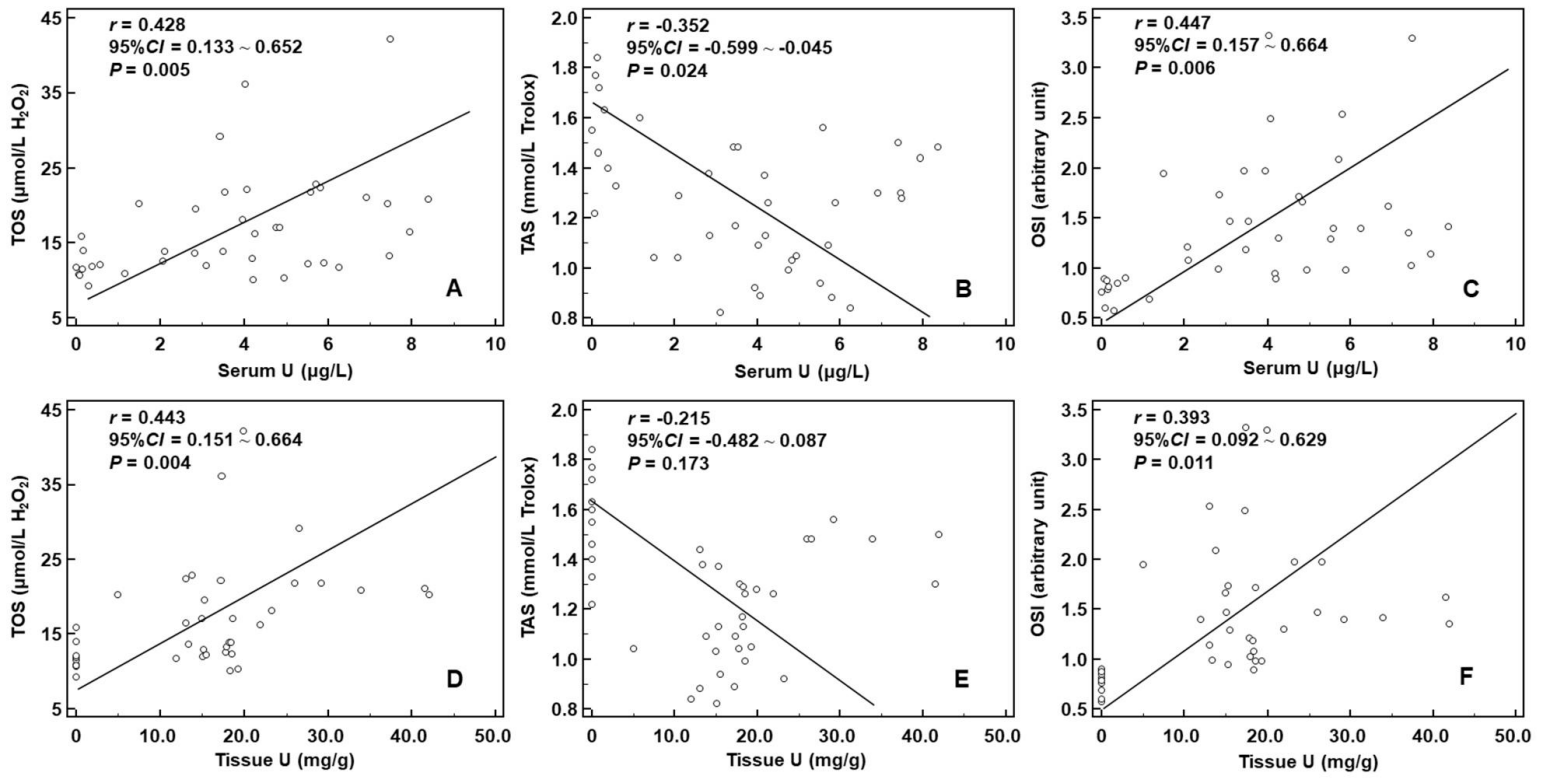
Correlation between tissue homogenate U (serum U) and oxidative stress parameters. *Notes* Tissue U content was positively correlated with TOS and OSI, but not with TAS. Serum U content was positively correlated with TOS and OSI, whereas it negatively correlated with TAS

We considered the values of different parameters for the NS group as the baseline levels and used Pearson correlation analysis to analyze the interrelationship of tissue/serum uranium and OxS parameters after uranium exposure. The results showed that a positive correlation existed between tissue homogenate U and serum U contents (*r* = 0.765; *P* < 0.001; Fig. 2). Tissue U content was positively correlated with TOS (*r* = 0.443; *P* = 0.004) and OSI (*r* = 0.393; *P* = 0.011), but not with TAS (*r* *=* −0.215; *P* = 0.173). Serum U content was positively correlated with TOS (*r* = 0.428; *P* = 0.005) and OSI (*r* = 0.447; *P* = 0.006), whereas it negatively correlated with TAS (*r* = −0.352; *P* = 0.024) (shown in Fig. 3).

### Serum levels of biomarkers of kidney injury and inflammation

Considering the NS group as a control, we observed the changes in serum biomarkers of kidney injury and inflammation after uranium exposure. The difference analysis showed that except for Hcy (*F* = 2.807; *P* = 0.059), the serum levels of kidney injury and inflammation biomarkers in the four groups of rats were significantly different (*F* = 5.402–12.106, *P* = <0.001–*P* = 0.006). Compared with the NS group (Table 2), the Lp-PLA2 level was significantly decreased in the U-24h (*t* = −3.429; *Padj* = 0.011), U-48h (*t* = 4.401; *Padj* = 0.002), and U-72h (*t* = 5.652; *Padj* < 0.001) groups. The CysC level was significantly increased in the U-24h (*t* = 3.482; *Padj* = 0.005), U-48h (*t* = 4.290; *Padj* = 0.003), and U-72h (*t* = 4.483; *Padj* < 0.001) groups. NGAL and CRP levels were significantly increased only in the U-48h (*t* = 3.013 and 2.911; *Padj* = 0.024 and 0.035) and U-72h (*t* = 3.785 and 5.564; *Padj* = 0.004 and <0.001) groups, whereas the Ure and Cre levels were significantly increased only in the U-72h (*t* = 4.310 and 5.156; *Padj* = 0.003 and <0.001) groups. Higher levels of Cre were observed in the U-72h group than in the U-48h (*t* = 3.419; *Padj* = 0.012) and U-24h (*t* = 5.227; *Pad*j < 0.001) groups. Higher levels of Ure (*t* = 3.552; *Padj* = 0.006) and CRP (*t* = 4.461; *Padj* < 0.001) were observed in the U-72h group.

**Table 2:** Kidney injury and inflammation biomarkers in the sera collected from the four groups of rats

Biomarkers	NS (n = 10)	U-24h (n = 10)	U-48h (n = 10)	U-72h (n = 10)	*F*	*P*
Ure (mmol/L)	4.11 ± 0.53	4.84 ± 1.17	6.13 ± 1.58	8.16 ± 3.58^a,b^	7.195	0.003
Cre (μmol/L)	21.5 ± 1.8	20.7 ± 3.6	36.7 ± 10.6	66.5 ± 37.3^a,b,c^	12.106	<0.001
CysC (μg/L)	0.76 ± 0.12	1.09 ± 0.35^a^	1.14 ± 0.15^a^	1.19 ± 0.22^a^	8.706	<0.001
NGAL (μg/L)	37.5 ± 14.7	91.3 ± 41.9	96.3 ± 56.6^a^	111.5 ± 47.0^a^	5.402	0.006
CRP (mg/L)	1.53 ± 0.55	2.25 ± 0.80	3.45 ± 0.97^a^	5.08 ± 2.40^a,b^	11.853	<0.001
Hcy (μmol/L)	9.62 ± 1.61	8.42 ± 1.97	7.96 ± 1.49	7.74 ± 1.33	2.807	0.059
Lp-PLA2 (IU/L)	861 ± 139	685 ± 116^a^	6448 ± 103^a^	585 ± 85^a^	11.366	<0.001

*Note* According to the POST HOC test of one-way ANOVA (Bonferroni-t test), ^a^ compared with the NS group, *Padj* < 0.05; ^b^ compared with the U-24h group, *Padj* < 0.05; ^c^ compared with the U-48h group, *Padj* < 0.05

**Fig. 4:**
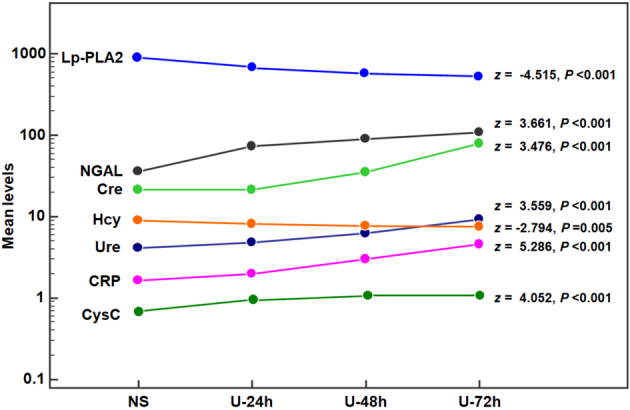
Variations in the kidney injury and inflammation biomarkers after the uranium exposure. *Notes* The measuring units are mmol/L for Ure, μmol/L for Cre and Hcy, μg/L for CysC and NGAL μg/L, mg/L for CRP, and IU/L for Lp-PLA2. The circular dot represents the mean of this biomarker in the same group. Except for Hcy, the variations in all biomarkers suggest that uranium exposure maybe induce the occurrence and development of kidney injury and tissue inflammation

The serum levels of kidney injury and inflammation biomarkers in the NS group were considered the baseline values in the Jonckheere–Terpstra test (Fig. 4). We found that the levels of kidney injury and inflammation biomarkers after uranium exposure showed time-dependent variations. The levels of Hcy (z = −2.794; *P* = 0.008) and Lp-PLA2 (z = −4.515; *P* < 0.001) were gradually decreased, whereas the levels of Ure (z = 3.559; *P* < 0.001), Cre (z = 3.476; *P* < 0.001), CysC (z = 4.052; *P* < 0.001), NGAL (z = 3.661; *P* < 0.001), and CRP (z = 5.286; *P* < 0.001) were gradually increased. These results suggested that uranium exposure induced the onset and development of kidney injury and tissue inflammation; however, the changes in the Hcy levels contradicted the increase in inflammation.

### Correlations between uranium exposure/OxS occurrence and kidney injury/inflammation

**Fig. 5:**
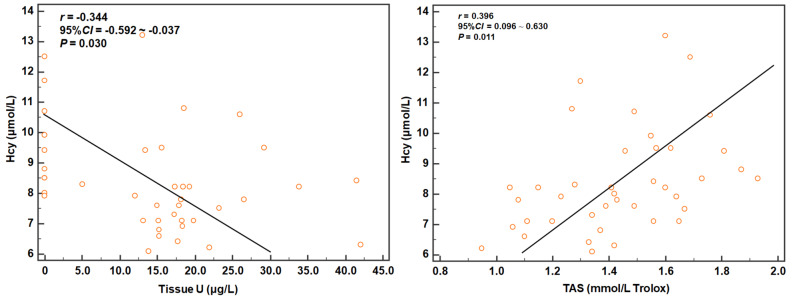
Correlation of Hcy with tissue U and TAS. *Notes* The relationship of Hcy with tissue U and TAS suggested that the depletion of antioxidants in the kidney tissues in a short span after uranium exposure may play an important role in the metabolism of Hcy

We analyzed the correlations between uranium/OxS parameters and kidney injury/inflammation using the Pearson correlation analysis (Table 3). Since the variations in Hcy were contrary to our assumption, we focused on the relationship between Hcy and uranium/OxS parameters (Fig. 5). All the significant correlations between the levels of kidney injury/inflammation biomarkers and the uranium levels/OxS parameters were mild to moderate. NGAL was negatively correlated with TAS (*r* = −0, 398; *P* = 0.011) and positively correlated with all the other parameters (*r* = 0.372–0.457; *P* = 0.003–0.018). On the contrary, Lp-PLA2 was positively correlated with TAS (*r* = 0.596; *P* < 0.001) and negatively correlated with all the other parameters (*r* = −0.414 to −0.493; *P* = 0.001–0.008). In addition, Ure and CysC were only positively correlated with Serum U (*r* = 0.377 and 0.501; *P* = 0.016 and 0.001). CRP was negatively correlated with TAS (*r* = −0.408; *P* = 0.009) and positively correlated with TOS and OSI (*r* = 0.431 and 0.457; *P* = 0.006 and 0.003). Hcy, our biomarker of focus, was negatively correlated with tissue U (*r* = −0.344; *P* = 0.030) and positively correlated with TAS (*r* = 0.396; *P* = 0.011). Taken together, the results suggested that OxS may be involved in the pathological process of kidney injury and tissue inflammation induced by uranium exposure. However, the relationship of Hcy with tissue U and TAS suggested that the depletion of antioxidants in the kidney tissues in a short span after uranium exposure may play an important role in the metabolism of Hcy.

**Table 3:** Pearson correlation coefficients among uranium levels/OxS parameters and kidney injury/inflammation biomarkers (n = 40; upper row: *r*; middle row: 95% *CI*; lower row: *t, P*)

Biomarkers	Tissue U	Serum U	TOS	TAS	OSI
Ure	0.109	**0.377**	0.206	−0.143	0.162
	(−0.210, 0.407)	**(0.075, 0.616)**	(−0.112, 0.487)	(−0.435, 0.177)	(−0.157, 0.451)
	0.675, 0.504	**2.511, 0.016**	1.300, 0.201	−0.890, 0.379	1.014, 0.317
Cre	0.019	0.301	0.214	−0.207	0.196
	(−0.294, 0.329)	(−0.012, 0.560)	(−0.105, 0.493)	(−0.487, 0.112)	(−0.123, 0.479)
	0.120, 0.905	1.943, 0.060	1.350, 0.185	−1.302, 0.201	1.234, 0.225
CysC	0.282	**0.501**	0.306	−0.223	0.278
	(−0.033, 0.545)	**(0.225, 0.703)**	(−0.006, 0.564)	(−0.500, 0.096)	(−0.037, 0.543)
	1.809, 0.078	**3.572, 0.001**	1.980, 0.055	−1.408, 0.167	1.784, 0.083
NGAL	**0.455**	**0.372**	**0.447**	**−0.398**	**0.457**
	**(0.167, 0.671)**	**(0.069, 0.613)**	**(0.157, 0.666)**	**(−0.631, −0.099)**	**(0.169, 0.673)**
	**3.148, 0.003**	**2.474, 0.018**	**3.077, 0.004**	**−2.672, 0.011**	**3.164, 0.003**
CRP	0.196	0.178	**0.431**	**−0.408**	**0.457**
	(−0.123, 0.479)	(−0.141, 0.464)	**(0.138, 0.656)**	**(−0.641, −0.109)**	**(0.170, 0.673)**
	1.235, 0.224	1.117, 0.271	**2.944, 0.006**	**−2.736, 0.009**	**3.170, 0.003**
Lp-PLA2	**−0.449**	**−0.414**	**−0.420**	**0.569**	**−0.493**
	**(−0.667, −0.160)**	**(−0.642, −0.117)**	**(−0.647, −0.125)**	**(0.313, 0.748)**	**(-0.698,** −**0.215)**
	**−3.096, 0.004**	**−2.801, 0.008**	**−2.856, 0.007**	**4.269, <0.001**	**−3.498, 0.001**
Hcy	**−0.344**	−0.197	−0.226	**0.396**	−0.322
	**(−0.592, −0.037)**	(−0.479, 0.122)	(−0.502, 0.092)	**(0.096, 0.630)**	(−0.566, 0.003)
	**−2.262, 0.030**	−1.240, 0.223	−1.430, 0.161	**2.658, 0.011**	1.996, 0.053

*Notes* Only NGAL and Lp-PLA2 were correlated with tissue U, serum U, and all OxS parameters

Significance values are presented in bold

### Close relationship of uranium exposure/OxS occurrence with kidney injury/inflammation

We took tissue/serum uranium content or OxS parameters as the dependent variables and used multiple linear regression to analyze the partial correlation coefficients and determine the close relationship of kidney injury/inflammation biomarkers with uranium exposure/OxS occurrence (Table 4). We only included either Ure or Cre because of the multicollinearity problem. Stepwise method analysis showed that NGAL level was closely correlated to the tissue U content (both *r*_*partial*_ = 0.455; *P* = 0.003) and CysC level was closely correlated to the serial U content (both *r*_*partial*_ = 0.501; *P* = 0.001) before and after adjusted analysis. The Lp-PLA2 level was closely correlated to all three OxS parameters, namely TOS (before adjustment *r*_*partial*_ = 0.365; *P* = 0.022 and after adjustment *r*_*partial*_ = 0.391; *P* = 0.014), TAS (both *r*_*partial*_ = 0.569; *P* < 0.001), and OSI (both *r*_*partial*_ = −0.494; *P* = 0.001). In addition, NGAL was also closely correlated to TOS (*r*_*partial*_ = 0.385; *P* = 0.016) before the adjusted analysis but the relationship was not observed after the adjusted analysis (*r*_*partial*_ = 0.232; *P* = 0.193). Overall, these results suggested that a close relationship existed between the uranium content in the tissue/blood and kidney injury, whereas OxS was closely related to endothelial inflammation.

**Table 4:** Partial correlation coefficients between kidney injury/inflammation biomarkers and uranium content or OxS parameters (n = 40)

Variables	Crude analysis			Adjusted analysis*		
	*r*_partial_	*t*	*P*	*r*_partial_	*t*	*P*
Tissue U						
Ure	−0.112	−0.645	0.523	−0.232	−1.305	0.202
CysC	0.177	1.034	0.309	0.190	1.057	0.299
NGAL	**0.455**	**3.148**	**0.003**	**0.455**	**3.148**	**0.003**
Lp-PLA2	−0.167	−0.972	0.338	−0.032	−0.177	0.861
CRP	−0.088	−0.509	0.614	−0.251	−1.419	0.166
Hcy	−0.076	−0.437	0.665	−0.227	−1.276	0.212
Serum U						
Ure	0.159	0.923	0.363	0.096	0.529	0.601
CysC	**0.501**	**3.572**	**0.001**	**0.501**	**3.572**	**0.001**
NGAL	0.147	0.855	0.399	0.014	0.078	0.938
Lp-PLA2	−0.116	−0.673	0.506	−0.014	−0.077	0.939
CRP	−0.114	−0.657	0.516	−0.218	−1.223	0.231
Hcy	−0.067	−0.388	0.700	−0.165	−0.915	0.368
TOS						
Ure	0.041	0.237	0.814	0.078	0.438	0.664
CysC	0.029	0.168	0.868	−0.016	−0.090	0.929
NGAL	**0.385**	**2.537**	**0.016**	0.232	1.330	0.193
Lp-PLA2	**0.365**	**2.388**	**0.022**	**0.391**	**2.586**	**0.014**
CRP	−0.129	−0.744	0.462	−0.082	−0.459	0.650
Hcy	0.112	0.649	0.521	0.139	0.783	0.440
TAS						
Ure	0.000	0.001	0.999	0.004	0.020	0.985
CysC	0.044	0.252	0.803	0.050	0.280	0.782
NGAL	−0.146	−0.846	0.404	−0.135	−0.757	0.455
Lp-PLA2	**0.569**	**4.269**	**<0.001**	**0.569**	**4.269**	**<0.001**
CRP	−0.169	−0.986	0.331	−0.171	−0.968	0.341
Hcy	−0.006	−0.035	0.973	−0.008	−0.045	0.965
OSI						
Ure	−0.002	−0.012	0.991	0.019	0.105	0.917
CysC	0.006	0.036	0.972	−0.023	−0.128	0.899
NGAL	0.279	1.667	0.105	0.226	1.290	0.207
Lp-PLA2	**−0.494**	**−3.498**	**0.001**	**−0.494**	**−3.498**	**0.001**
CRP	0.283	1.694	0.100	0.299	1.746	0.091
Hcy	0.054	0.311	0.758	0.071	0.394	0.696

*Note* *Adjusted by the OxS parameters (if uranium content was the dependent variable) or by tissue/serum uranium content (if OxS parameter was the dependent variable). All the significant partial correlation coefficients were derived from the results of the Stepwise method, and no-significant partial correlation coefficients were derived from the results of the Enter method

Significance values are presented in bold

### Discussion

Uranium is considered a rare metal, and its content in the earth’s crust is much higher than that of elements such as mercury, bismuth, and silver. People living in uranium-rich environments are vulnerable to adverse effects of uranium, including injury to some tissues and organs leading to serious diseases. According to relevant research reports on the Persian Gulf syndrome, kidneys are the primary target organ for the chemical toxicity of uranium [26]. Uranium exposure may cause kidney cell toxicity through multiple signaling pathways to suppress cell viability and induce apoptosis. This kidney cytotoxicity may be related to uranium-induced enhancement in the reactive oxygen species production [27], reduction of mitochondrial membrane potential [19], and inflammatory response [28]. Although the kidney chemical toxicity of uranium has been studied in the past two decades, many unresolved issues still exist. The previous determination of the occurrence of uranium-induced OxS is based on changes in some or several oxides or antioxidants, and cannot fully reflect the true state of OxS in the body. Here, we evaluated the oxidative and antioxidant systems of the rats to understand their relationship to uranium accumulation in the kidneys and blood, as well as their relationship to kidney injury and inflammation. We found that while uranium content in the kidneys decreased over time, uranium level in the blood decreased at 48 h and slightly rose again at 72 h, which indicated that the kidney excretion of uranium was not the only way to clear uranium, and the reabsorption through kidney tubules may also play an important role. The serum uranium was not continuously decreased but increased at 72 h, and was lower than that at 24 h, which could potentially be attributed to a combination role of uranium filtration from the glomeruli and reabsorption in the renal tubules. During this process of uranium change, OxS continued to worsen without uranium dose-dependent relationship. Aditionally, we also found that tissue uranium content was only closely related to NGAL (a marker of kidney injury), serum uranium content was closely related to only CysC (an ideal marker of glomerular filtration function), and the OxS parameters (TOS, TAS, and OSI) were closely related to only Lp-PLA2 (a marker of endothelial inflammation). These results indicated that the detection of uranium content at different sites may have different clinical significance, and the pathogenesis of kidney injury caused by OxS may be related to the occurrence and development of endothelial inflammation. Notably, the Hcy levels were not elevated and a decrease in its level was observed after uranium exposure. We speculated that this phenomenon might be related to Hcy as a raw material for methionine metabolism.

Here, we clarified several questions about the mechanism of the kidney chemical toxicity of uranium. First, our results confirmed that the occurrence of uranium-induced OxS was not only limited to the tissue [29, 30] or cellular [31, 32] level, but also occurred at the level of the overall oxidation and antioxidant systems of the body. The difference is that significant changes in oxides and antioxidants at the tissue or cellular level often occur within 24 h of uranium exposure [29–32], but significant changes in TOS, TAS, and OSI occurr between 24 and 48 h with a slight delay. Second, it has reported that no direct evidence indicates whether nephrotoxic injury from uranium was dominated by the direct effects of uranium chemotoxicity or indirectly through the OxS pathway [33–35]. However, our analysis of the changes in the OxS parameters, kidney injury, and inflammation from a time series perspective revealed that when rats did not exhibit significant OxS within 24 h, there were notable changes in CysC and Lp-PLA2 levels. This suggests a direct toxic effect of uranium during kidney injury. Notably, we found that regardless of whether the OxS parameters were corrected or not, uranium content in the tissue was only closely related to NGAL (a marker of kidney injury, especially renal tubular injury [36, 37]). This observation suggested the direct chemical toxicity of uranium during kidney injury. Further, before adjusting for uranium content, Lp-PLA2 (a marker of endothelial inflammation [38, 39]) and NGAL were closely related to TOS; however, after adjusting for uranium content, only Lp-PLA2 was closely related to TOS, and the close relationship between NGAL and TOS disappeared. These results also indirectly suggested that kidney injury (especially renal tubular injury) may be greatly affected by the correction factor i.e., uranium content, and the direct chemical toxicity of uranium may play a dominant role in the process of kidney injury, whereas the uranium-induced OxS injury was closely related to endothelial inflammation. In addition, the markers of kidney injury that were closely related to tissue and serum uranium levels were not the same. The close relationship between serum uranium and CysC suggested that the quantification of serum uranium may indicate the risk of glomerular injury, possibly associated with the excretion of uranium in the blood through glomerular filtration. Therefore, understanding changes in serum uranium levels and the OxS parameters during acute or long-term chronic uranium exposure may assist in the risk management of kidney injury after uranium poisoning.

Interestingly, Hcy is a marker of endothelial inflammation [38] and a potential independent risk factor for chronic kidney disease and acute kidney injury [40, 41]. We anticipated that the Hcy levels should gradually increase with the occurrence and development of uranium-induced kidney injury and endothelial inflammation. Recently, Shyamkrishnan et al. showed that the increased Hcy levels was related to the OxS occurrence during kidney injury [42]. However, our results indicated a decrease in the serum Hcy level, which was positively correlated with the decrease in TAS after uranium exposure. Considering that Hcy is one of the important raw materials for methionine metabolism and glutathione synthesis [43] and the uranium-induced OxS decreases glutathione levels [44], we speculated that the variations in the Hcy levels might be the result of glutathione continuous synthesis against the OxS. Therefore, the changes in Hcy after 72 h of uranium exposure may be a short-term effect which tried to achieve the oxidation-antioxidation balance. However, an elevated Hcy reported in several literatures may be the final effect of the long-term interaction between kidney injury [45], oxidative stress [42], and Hcy metabolism [46], and the mechanism is still unclear in nephropathy patients.

### Conclusion

Our findings determined that uranium exposure can cause kidney tissue and inflammatory injuries through direct chemical toxicity or an OxS-mediated pathway. The direct chemical toxicity of uranium was more closely related to renal tubular injury, whereas the uranium-induced nephrotoxic oxidative stress was closely associated with subsequent endothelial inflammation. Further, we clarified preventive strategies for the risk management of kidney injury after uranium poisoning and enlisted potential strategies for treating uranium poisoning. However, our study has some limitations because the variations in different parameters were not determined in the same animal after 24, 48, and 72 h of uranium exposure. This happened because rats have to be executed after each time point because of the requirement of enucleating kidney tissue.

## Data Availability

The datasets used and analyzed during the current study are available from the corresponding author upon reasonable request.
